# Homeobox Genes in Cancers: From Carcinogenesis to Recent Therapeutic Intervention

**DOI:** 10.3389/fonc.2021.770428

**Published:** 2021-10-14

**Authors:** Yangyang Feng, Tongyue Zhang, Yijun Wang, Meng Xie, Xiaoyu Ji, Xiangyuan Luo, Wenjie Huang, Limin Xia

**Affiliations:** ^1^ Department of Gastroenterology, Institute of Liver and Gastrointestinal Diseases, Tongji Hospital of Tongji Medical College, Huazhong University of Science and Technology, Wuhan, China; ^2^ Hubei Key Laboratory of Hepato-Pancreato-Biliary Diseases, Tongji Hospital, Tongji Medical College, Huazhong University of Science and Technology, Wuhan, China; ^3^ Hepatic Surgery Center, Tongji Hospital, Tongji Medical College, Huazhong University of Science and Technology, Wuhan, China

**Keywords:** homeobox genes, transcription factors, therapy, biomarker, cancer progression

## Abstract

The homeobox (HOX) genes encoding an evolutionarily highly conserved family of homeodomain-containing transcriptional factors are essential for embryogenesis and tumorigenesis. HOX genes are involved in cell identity determination during early embryonic development and postnatal processes. The deregulation of HOX genes is closely associated with numerous human malignancies, highlighting the indispensable involvement in mortal cancer development. Since most HOX genes behave as oncogenes or tumor suppressors in human cancer, a better comprehension of their upstream regulators and downstream targets contributes to elucidating the function of HOX genes in cancer development. In addition, targeting HOX genes may imply therapeutic potential. Recently, novel therapies such as monoclonal antibodies targeting tyrosine receptor kinases, small molecular chemical inhibitors, and small interfering RNA strategies, are difficult to implement for targeting transcriptional factors on account of the dual function and pleiotropic nature of HOX genes-related molecular networks. This paper summarizes the current state of knowledge on the roles of HOX genes in human cancer and emphasizes the emerging importance of HOX genes as potential therapeutic targets to overcome the limitations of present cancer therapy.

## 1 Introduction

### 1.1 General Overview

The homeobox (HOX) genes were initially discovered in 1992 with the roles in embryogenesis in *Drosophila melanogaster*, where mutations in antennapedia and bithorax clusters were observed to lead to abnormal body development (1). These mutations lead one body segment to emerge similar to another segment and is originally called the term “homeotic” mutations since the 1990s (2).

The HOX genes encode a family of transcription factors that regulate embryogenesis and morphogenesis during the embryonic period and adulthood. These genes are highly conserved and contain homologous domains in almost all eukaryotic cells (3). The HOX genes comprise a highly conserved sequence of 180-183 base pairs encoding a homeodomain of 60 or 61 amino acids helix-turn-helix motif (2). The specific structure was initially characterized by magnetic resonance spectroscopy imaging.

**Figure 1:**
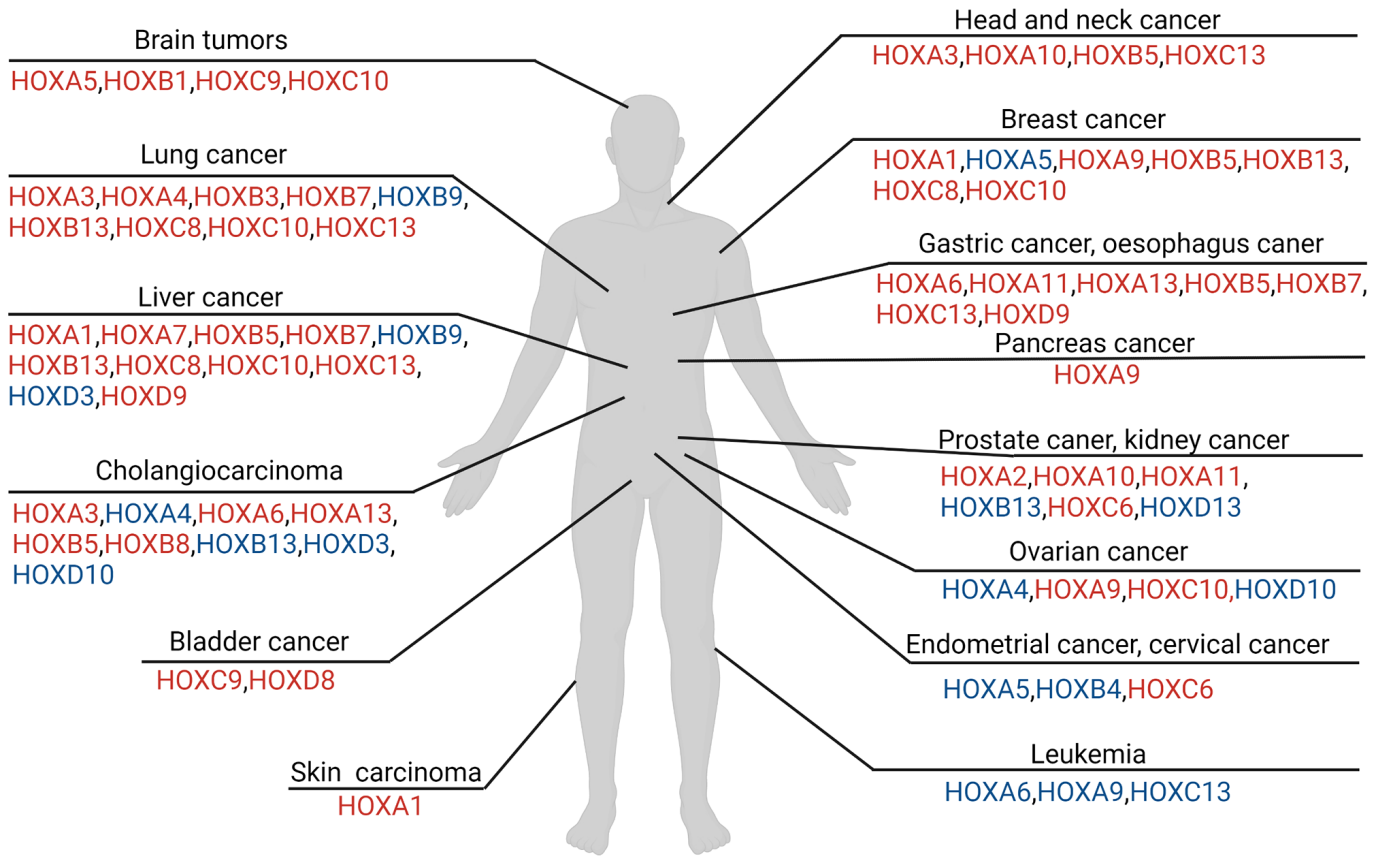
Overview of HOX involvement in different tumors in human. The colors of HOX molecules indicate regulations in relevant tumor cell. The red font means the corresponding HOX genes is up-regulated in tumor, while the blue font means the corresponding HOX genes is down-regulated in comparison. Regulation of HOX factors in various tumor types described in this figure legend. [brain cancer mainly includes the glioma and neuroblastoma (4–11), lung cancer (12–24), hepatocellular carcinoma (25–31), bladder cancer (32, 33), cholangiocarcinoma (34), head and neck squamous cell carcinoma (35, 36), breast cancer (21–23, 37–54), gastric carcinoma and esophagus cancer (55–64), prostate cancer and renal carcinoma (43, 65–72), ovarian cancer (73–79), endometrial cancer and cervical cancer (80–84), leukemia (85–95), skin carcinoma (96–98)].

In the human genome, according to the sequence similarity and position correlation in the chromosome, 39 HOX family genes can be divided into 4 clusters, namely HOXA, HOXB, HOXC, HOXD. The number after the subgroup HOXA, HOXB, HOXC, HOXD increases orderly in a 3’ to 5’ orientation, which is shown in **Figure 1**. Each cluster has between 9 and 11 genes in a row on a homologous strand of DNA, which contains duplication and divergence of ancestral HOX genes.

### 1.2 Physiological Function of HOX Genes

HOX genes encode a family of master transcriptional regulators throughout growth and development in human tissues and organs. This period monitoring elicits distinct and temporospatial limb and organ developmental programs along the anterior-posterior axis (99). Researchers found the chromosomal arrangement of HOX genes was closely related to their localization order of genetic expression (100).

Plenty of scientific evidence suggests that the specific functions of individual HOX genes largely reflect their regional and restricted expression patterns. The disruption of the chromosome region related expression pattern may lead to developmental defects and diseases, especially human cancer (101).

HOX proteins encoded by HOX genes are key components of substantial metabolic processes such as lipidic metabolism, and their roles in organogenesis and tumorigenesis have been studied in detail over the past decade (12, 37, 73, 102–105). Pluripotent embryonic carcinoma cells have been associated with the differentiation of a broad spectrum of tissues early in development in mice embryos (106). This process appears to be related to retinoic acid reactions, the chromosomal region determining the aforementioned relation was regulated by HOX gene (96, 107).

In conclusion, HOX genes act as primary regulators to regulate downstream target molecules (103). HOX genes play an essential role in embryonic development, including morphogenesis, organogenesis, and differentiation of a variety of tissues and organs (108).

## 2 Deregulation of HOX Genes in Human Cancer

Over the past several decades, we have come to understand that a great many genes and proteins controlling embryogenesis usually partake indispensable effects in carcinogenesis likewise. Many genes identified as pivotal genes in carcinogenesis, for example, oncogenes or tumor suppressor genes, have been discovered to paly primary roles in embryogenesis correspondingly. It indicates both processes are tightly linked (109). The familiar components of signal transduction pathways, such as the Sonic Hedgehog pathway, Wingless-Type MMTV Integration Site Family, Notch pathway, Paxillin pathway, and Sry-box transcription factors family members, closely act in accordance with the above rules.

**Figure 2:**
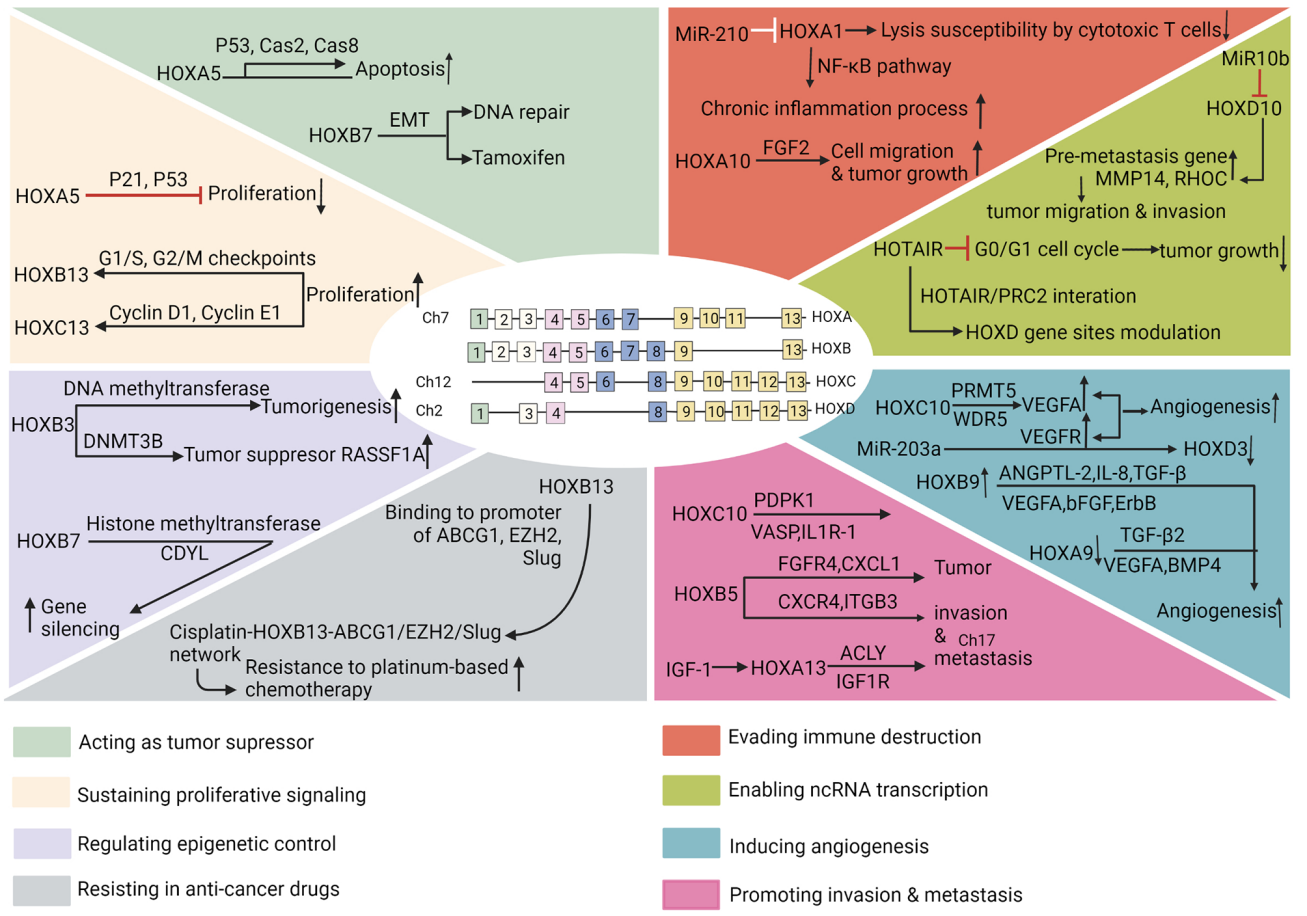
HOX genes in the hallmarks of cancer. The hallmarks of cancer comprise eight biological capabilities acquired during multistep development of human tumors. The hallmarks constitute an organizing principle for rationalizing the complexities of neoplastic disease. They include acting as tumor suppressor, sustaining proliferative signaling, regulating epigenetic control, resisting in anti-cancer drugs, evading immune destruction, enabling ncRNA transcription, inducing angiogenesis, and promoting invasion and metastasis.

This close relationship between embryogenesis and carcinogenesis supports the lineage-dependency theory. The theory proposes that the cellular mechanisms participating in lineage heredity and adaptability during development, potentially underlies tumorigenic mechanisms in humans (110). The HOX genes might be applicable to the lineage-dependency theory. Apart from the indispensable involvement of HOX genes in embryonic development in physiological status, their abnormal expression, has long been linked to tumors. Initially, these genes were thought to be related to human cancers in the hematologic system and embryo. Later, many other kinds of tumors were also noticed to be associated with deregulated HOX genes (85, 101, 111). In the context of cancer development, the abnormal expression of HOX gene may affect cell proliferation, differentiation, apoptosis, motility, angiogenesis, autophagy, and cell receptor signaling (112). HOX genes in the hallmarks of cancer are shown in **Figure 2**.

The protein products of HOX genes are initially thought to be transcription factors that accelerate cancerization since HOX proteins are deregulated in carcinogenesis. In the above process, HOX proteins govern the intricate balance of multiple signaling pathways on and off, and affect downstream targets of these pathways (113), so as to determine different cancer outcomes.

Subsequent studies have shown that HOX genes act not only as transcriptional activators but also as transcriptional repressors in cancer. This abnormal regulation of HOX genes in cancer suggests that HOX genes expression is an integral part of the regulatory network (103, 114, 115).

HOX genes in tumor show temporospatial deregulation pattern, different from that in normal tissues and organs. Besides, the gene dominance in expression level, that is, the aberrantly increased expression level of HOX genes in specific tissue types, the mechanism is also proposed to explain HOX genes relevant to cancer. The targets identified for HOX factors in human cancer are shown in **Table 1** (4–7, 13–20, 25–30, 32–35, 38–49, 55–62, 65–70, 74–76, 80–83, 86–91, 116–121, 127–130, 133–149).

**Table 1:** Targets identified for HOX factors in human cancer.

HOX protein	Role	Interference	Cancer type	Year	Reference
HOXA1	Oncogene	Sequester G9a/EZH2/Dnmts, sponge miR-193a-5p, via cyclin D1, *via* miR-100	Breast cancer, glioma, GC, lung cancer	2014,2016,2018	(38, 63, 97, 116)
HOXA3	Oncogene	Upregulate methylation level, promote differentiation to angiogenesis, confer cisplatin resistance	NSCLC, PTC, blood	2011,2019	(13, 117, 118)
HOXA4	Tumor suppressor	Downregulate β-catenin, Cyclin D1, c-Myc and survivin, indicate inhibition of Wnt signaling, upregulate GSK3β	Lung cancer, ovarian carcinoma	2009,2018	(14, 74, 79)
HOXA5	Tumor suppressor	Induce apoptosis mechanism mediated by cas2 and cas8; regulate E-cadherin and CD24; methylate promoter region & limit p53 expression	Breast cancer, mammary cancer, cervical cancer	2015-2021	(9, 21, 39–41, 50, 51, 80, 84, 119–126)
HOXA6	Oncogene	Coexpress with PBX2	GC, CRC, leukemia	2021	(55)
HOXA7	Oncogene	Combine to Snail promoter, cyclin E1/CDK2, activate Snail	Cervical cancer, HCC	2016,2020	(25, 81)
HOXA9	Oncogene	Pioneer factor at *de novo* enhancers and recruit CEBPα & MLL3/MLL4 complex, regulate BRCA1, activate JAK/STAT, induce 1GF1R expression	Pancreatic cancer, leukemia, NSCLC	2017-2020	(75, 77, 86–90, 92, 94, 127–132)
HOXA10	Oncogene	Suppress FASN transcription by forming a protein complex with AR and prevent AR recruitment to FASN gene promoter, hinder mir195	Prostate cancer, testicular cancer, HNSCC	2020	(35)
HOXA11	Oncogene	LncRNA HOXA11-AS recruit EZH2 along with the histone demethylase LSD1 or DNMT1	GC, LUAD, renal cancer	2016,2017,2018	(56, 57)
HOXA13	Oncogene	IGF-1	CRC, GC	2021	(133)
HOXB4	Tumor suppressor	Downregulate activity of Wnt/β-catenin signaling pathway, downregulate P-gp, MRP1 and BCRP expression	Cervical cancer, leukemia	2016,2021	(82, 91)
HOXB5	Oncogene	Transactivate CXCR4, ITGB3, FGFR4, CXCL1	HCC, CRC, breast cancer, HNSCC, GC	2015,2021	(26, 42, 134)
HOXB7	Oncogene	Reprogram to iPSC with comparable efficiency to LIN28B or c-MYC, activate TGFβ signaling pathway	Lung cancer, GC	2016,2018	(15, 27, 34, 58, 135, 136)
HOXB8	Oncogene	Instigate BACH1-mediated transcriptional cascade, viaZEB2 targets	GC, CRC	2017,2020	(59, 137)
HOXB13	Tumor suppressor	Suppress C-Myc expression to exert antitumor effects *via* β-catenin/TCF4 signals, network with ABCG1/EZH2/Slug	Colon cancer, lung cancer, prostate cancer	2015-2019	(16, 43–46, 60, 65–68, 138–141)
HOXC6	Prognosis marker, oncogene	Enhance BCL2-mediated antiapoptotic effects, drive MET	Prostate cancer, cervical cancer,HCC	2019	(28, 69, 83)
HOXC8	Oncogene	Upregulate TGFβ1, repress LMP1	NSCLC, TNBC	2018	(17, 47, 48, 142, 143)
HOXC9	Oncogene	Mediate autophagy, *via* mir-495/HOXC9 axis, promote multi-chemoresistance	Bladder cancer, neuroblastoma, NSCLC	2011,2015,2016	(4, 5, 32)
HOXC10	Oncogene	*Via* upregulating PDPK1, VASP, EMT, promote angiogenesis, induce immunosuppressive gene	HCC, lung cancer, ovarian cancer	2014-2020	(6, 7, 19, 29, 49, 61, 144, 145)
HOXC13	Oncogene	Modulate CCND1 & CCNE1	Lung adenocarcinoma	2017	(20)
HOXD3	Tumor suppressor	Inhibit HDAC1 *via* ITGA2 pathway & MAPK/AKT signaling	HCC, CRC	2019,2020	(146, 147)
HOXD8	Oncogene	Enhance LINC01116 contribution to progression of BCa *via* targeting ELK3 & HOXD8	Bladder cancer	2020	(33)
HOXD9	Oncogene	Transactivate RUFY3 & ZEB1, promote MET	GC, HCC	2015,2019	(30, 62)
HOXD10	Tumor suppressor	Inhibit RHOC/AKT/MAPK pathway, upregulate mir-10b	CRC	2019	(76, 148)
HOXD13	Tumor suppressor	Inhibit SMAD1, suppress BMP4	Prostate cancer	2021	(70)

### 2.1 Direct Role: As Oncogenes

The HOX genes have been reported to act as oncogenes and contribute to tumor progression in several types of cancer. For example, simultaneous overexpression of HOXA9 and MEIS1A induces acute myeloid leukemia in rats (92, 131). In breast cancer, HOXB7 has been reported as an oncogene because its upregulation appears to promote the expression of bFGF and induce the epithelial-mesenchymal transitions (EMT) (15, 27, 34). In colorectal cancer, HOXB5 overexpression mediated by CXC chemokine ligand 12 facilitates metastasis through transactivating downstream protein CXCR4 and ITGB3 (134). HOXB13 seems to be an oncogene for ovarian and prostate cancer. Its knockout in ovarian cancer cells results in reduced tumor invasion (45, 65, 71, 138, 141). On the contrary, its ectopic expression promotes cell proliferation and non-anchoring. Mutations and growing resistance to tamoxifen-mediated apoptosis in the cell tumor antigens *P53, Myc, and Ras* are included in mouse ovarian cancer cells (43–46). In addition, HOXB13 overexpression appears to promote invasion of these types of cancers. In prostate cancer, HOXB13 regulates the prostate-derived ETS family members and also facilitates cell invasion (140). As HOXB3 in glioblastoma, its knockout leads to cancer cell suppression (150). In squamous carcinoma of the cervix, HOXC10, which is overexpressed and thought to be an oncogene, is knocked out to reduce invasiveness (6, 19, 29, 61). In colorectal cancer, HOXA13 overexpression mediated by insulin-like growth factor 1 promotes metastasis through upregulating downstream targets ATP-citrate lyase and insulin-like growth factor 1 receptor (133). Ectopic overexpression of ATP-citrate lyase and insulin-like growth factor 1 receptor rescues the decreased colorectal cancer metastasis induced by HOXA13 knockdown (133). HOXC10 overexpression mediated by Interleukin 1β facilitates hepatocellular carcinoma (HCC) metastasis (29). The above studies implicate HOX genes as oncogenes and prognostic biomarkers. Hence, targeting HOX genes’ relevant pathways is likely to be the promising therapeutic option for clinical cancer prevention (29).

### 2.2 Direct Role: As Tumor-Suppressor Genes

Basing on our review of the literature, we found the expression of specific HOX genes in cancers tends to vary with tissue type and tumor sites. Besides, the HOX genes have been found to behave as tumor-suppressor genes and dedicate to tumor suppression in several cancers.

HOXC8 in nasopharyngeal cancer is silent and its ectopic expression causes the inhibition of tumor growth (143). *In vitro* and *in vivo*, in addition, HOXA5 is often down-regulated in breast cancer and appears to mediate apoptosis through p53 or caspases 2 and 8 in normal cells, thus having a tumor-like effect inhibition (50, 121, 122). Downregulation of HOXC9 in infant neuroblastoma appears to lead to increased cancer cell survival and tumor growth (5). HOXB1 is also significantly down-regulated in glioma cells, its knockdown promotes proliferation and invasion, inhibits apoptosis of cancer cells *in vitro*, and leads to poor survival (5, 8, 151).

The expression changes of HOXA4, HOXA9, and HOXD10 cause abnormal proliferation and differentiation of colorectal carcinoma cells and contribute to tumor development (14, 74, 87, 127). In addition, HOXB3 overexpression promotes the proliferation and invasion of glioblastoma cells, acute myeloid leukemia, pancreas, prostate, ovarian, and lung cancer (74, 93, 150, 152, 153). Excessive HOXB7 inducing MET in breast cancer cells also interferes with DNA repair and tamoxifen (136). HOXA5 down-regulation is discovered in multiple tumors, including liposarcoma, cervical cancer, breast cancer, which suggests that HOXA5 may be an important tumor suppressor (21, 51, 84, 123). The promoter methylation and downregulation of HOXA5 during the epigenetic deregulation decrease RARβ-driven apoptosis mediated by caspase 2 and caspase 8. Therefore, HOXA5 consumption in breast cells causes a lack of epithelial cell characteristics as well as an increase of stem cell property and cellular plasticity, eventually leads to a more aggressive phenotype (119).

Therefore, the abnormal expression of the HOX genes in various cancers seems to indicate that random alterations are not necessary for the health maintenance and survival of cancer cells. Instead, abnormal expression of specific HOX genes in cancer seems to play a large part in the development of cancer.

However, HOX genes may show a predisposition. It is up-regulated or down-regulated in certain tumors, promoting cancer progression or inhibition in certain cases. Thus, they can be regarded as oncogenes or tumor suppressor genes, usually depending on the corresponding tumor microenvironment.

### 2.3 Indirect Role: Epigenetic Control *via* HOX Genes

HOX proteins are also involved in chromatin posttranslational modifications, epigenetically affect the expression of crucial cancer progression genes. For example, HOXB3 regulates the expression of DNA methyltransferase, which can add methyl to the 5 positions of cytosine ring carbon in CpG dinucleotide (93, 152, 153). Therefore, HOX proteins can indirectly regulate the expression of other genes by controlling the expression of methyltransferase. Thus, the HOX genes may have an indirect effect on cancer by controlling the expression of DNA methyltransferase, which seems to be of considerable relevance to understanding the mechanisms involved in tumorigenesis.

In fact, all cancer progression has common abnormalities, such as the massive epigenetic silencing of tumor suppressor genes. The mechanisms behind this phenomenon are remained to be fully understood. The DNA methyltransferase protein family consists of DNA methyltransferase (DNMT)-1, DNMT3A, DNMT3B, and DNMT3L (154). The last is associated with *de novo* methylation of DNMT3A/B, which is required during embryonic development, imprinting, and X chromosome inactivation. DNMT1 maintains the DNA methylation profile of the genome during each cell division. DNMT3A and DNMT3B can actively add *de novo* methyl to DNA sequences to control gene expression and play a key role in cancer progression.

Palakurthy has shown that DNMT3B is the target of HOXB3 protein and is involved in the epigenetic regulation of tumor suppressor gene RASSF1A in lung adenocarcinoma and other cancers (153). HOXB3 also directly interacts with DNMT3B to promote the occurrence of leukemia in acute myeloid leukemia.

In addition, HOX genes may also play a role in post-translational modification of chromatin and affect gene expression. For instance, Heinonen has shown that HOXB7 can directly bind to chromodomain protein Y-like and enhance the histone methyltransferase activity of Polycomb Complex 2 and induce gene silencing as typical for trimethylate lysine 27 of histone H3 (155).

### 2.4 Tumor Proliferation, Invasion, and Metastasis

Proliferation phenotype has been found in various HOX-related researches, especially in leukemia, where HOX upregulated expression is often resulted from translocation mutations or altered regulation in the trithorax homologue myeloid-lymphoid leukemia (112). Invasion and metastasis phenotypes caused by abnormal HOX gene expression have been mostly studied in solid tumors, where HOX gene deregulation is usually due to loss-of-function mutations or gain-of-function mutations, or undefined mutations in upstream regulators.

In recent work, HOXC13 promotes cell proliferation by modulating the expression of cyclin D1 and cyclin E1 in lung adenocarcinoma. HOXA5 inhibits cell proliferation by regulating p53 expression in liposarcomas and p21 in non-small lung cancers (20, 63, 84, 120). Although HOXB13 overexpression promotes prostate cancer metastasis by downregulating intracellular zinc and upregulating NF-kappaB signaling pathways, HOXB13 consumption promotes proliferation of PC-3 and LNCaP cells by controlling G1/S and G2/M checkpoints (16, 60, 67, 138, 141).

HOX proteins affect cell cycle process by regulating cell cycle-related proteins (156), thus also affect proliferation and apoptosis in cancer progression. Growing evidence is noticeable that many HOX transcription factors are abnormally expressed in cancer, and their dysregulation significantly promotes tumor invasion and metastasis.

HOXD9 interacts with the promoter region of zinc-finger E-box binding homeobox (ZEB)-1, inhibition of ZEB1 induced by HOXD9 suppresses HCC cell migration, invasion as well as EMT (30). In addition, according to microarray analysis, ZEB2 may be a downstream cofactor of HOXB8 (59). Patients enrolled in studies have shown that high HOXB13 expression promotes the progression of lung adenocarcinoma and predicts poor prognosis (16).

In epithelial ovarian cancer cells, HOXA9 not only promotes the growth of epithelial ovarian cancer cells *in vivo* by activating transcriptional activity of the gene encoding transforming growth factor β (TGFβ)-2, but also binds to the promoter of the cadherin3 gene that encodes P-cadherin to induce intraperitoneal dissemination (77, 94). Many reports suggest the temporospatial deregulation of HOXA9 is associated with primary tumors and specific histological subtypes. HOXA9 is of therapeutic potency, while the potency is limited by the low membrane permeability.

### 2.5 Angiogenesis

Angiogenesis is an important link during tumor progression. HOX proteins affect angiogenesis mainly by regulating VEGF expression (7). For example, HOXC10 level is statistically correlated with VEGFA expression in gliomas. HOXC10 upregulates VEGFA expression transcriptionally by binding to its promoter, and the post-translational modification of histones mediated by protein arginine methyltransferase 5 and WD repeat domain 5 is required in angiogenesis (7).MiR-203a negatively targets HOXD3 directly by targeting the VEGFR promoter region and increases VEGFR expression.

HOX protein expression also plays an important role in endothelial cells (EC) (146). For instance, HOXA5 is expressed in static EC but not in activated angiogenic EC. HOXA5 continuously increases the TSP-2 expression and decreases the VEGF expression, thereby inhibiting histopathological angiogenesis (124, 125). In addition, HOXA5 is absent in EC of proliferative hemangioma (9). HOXA5 increases the mRNA and protein expression of Akt1, further enhances Akt activity by coordinating the down-regulation of PTEN, thereby increasing the stability of the capsular patellar junction (126).

### 2.6 Resistance in Anti-Cancer Drugs

HOX proteins are involved in resistance to the anti-cancer drugs in cancers, especially in myeloid leukemia, HCC, breast cancer, and lung cancer. HOX proteins regulate various non-coding RNAs (ncRNAs) which influence cancer cells chemotherapy resistance. In particular, we take the example of the role of HOXB13 in mediating chemotherapy resistance in lung adenocarcinoma. HOXB13 upregulates a series of drug-transfer and drug-resistance-related genes by directly binding to their promoters and forming the cisplatin-HOXB13-ABCG1/EZH2/Slug network, including *ATP Binding Cassette Subfamily G Member 1*, *Enhancer Of Zeste 2 Polycomb Repressive Complex 2*, and *Slug (* 16, 139). Levels of HOXB13 and its target genes may help predict sensitivity of platinum-based chemotherapy in lung adenocarcinoma patients (16).

### 2.7 HOX-Mediated Molecular Crosstalk During Tumorigenesis

#### 2.7.1 Post-Translational Modifications

A variety of signaling pathways that regulate proliferation, apoptosis, differentiation, movement, and angiogenesis, interact with HOX transcription factors family. Post-translational modifications of HOX protein also play a key role in tumorigenesis. HOX post-translational modifications are an under-valued project. In most eukaryotic proteins, the turn-over, intracellular localization, molecular interactions and activity are modulated by post-translational modifications. The post-translational modifications of HOX proteins in cancer mainly contributes to the modulation of protein stability, DNA binding, transcriptional activator-like effectors interaction, transcriptional activation capacity, unidentified cellular impact (157).

#### 2.7.2 MicroRNA and Non-Coding RNA

Of the 39 HOX genes, 30 HOX genes contain more than one conserved nucleotide sequences that are expected to be targets of miRNAs in vertebrate (2). We found that the HOX targeting genes are mainly located on the 3’ side of each HOX miRNA site. This study shows that HOX miRNAs help inhibiting abundant pre-gene expression, hence enhancing the prevalence of post-genes. In patients with gastrointestinal stromal tumors, the level of miRNA196a is positively related to higher tumor histologic grade, higher recurrence rate, and lower survival rate (64).

In addition, HOX transcribed antisense RNA (HOTAIR) also contributes to HOX expression regulation. HOTAIR is a 2.2-kilobase long non-coding RNA that is transcribed from the antisense strand of HOXC clusters, and its function is to inhibit the transcription of 40 kilobases of HOXD clusters (158, 159). The mechanism of action of these long non-coding RNAs has not been fully determined. However, HOTAIR has been shown to regulate chromatin state and kinetics by binding to specific chromatin modification complexes. The trimethylation of histone H3 lysine 27 at the HOXD site requires HOTAIR/PRC2 interactions. Knocking out HOTAIR activates HOXD gene transcriptional activity on human chromosome 2 (158, 160, 161). Thus, it is possible to modulate the HOXD gene by modulating HOTAIR levels, which has implications in cancer therapy. For example, the expression of HOTAIR is closely related to the neoplasm staging and poor prognosis of glioma. Reducing HOTAIR expression induces inhibition of colony formation, G0/G1 cell cycle arrest, and inhibition of tumor growth *in situ (* 10). Thus, HOTAIR seems to serve as a prognostic factor for survival, as well as a biomarker for identifying molecular subtypes in cancer (162).

Recent evidence underscored the function of long noncoding RNAs (lncRNAs)-driven hepatocarcinogenesis. The expression level of lncRNA HOXA transcript at the distal tip (HOTTIP) and HOXA13 is associated with metastasis and survival in HCC patients. It indicates the prospective potency of HOTTIP and HOXA13 to be the predictive biomarker in HCC (163).

Summing up the above, we highlight the specific roles of miRNAs and lncRNAs in human cancer. They perform not only as the intermediary between DNA and protein, including chromosome remodeling, transcription, and post-transcriptional processing, but as leading characters in body balance adjustment during congenic malformation, oncogenesis, metabolic processes, and deregulation of cell cycle (164).

## 3 HOX Transcription Factors as Therapeutic Targets

### 3.1 HOX Genes Act as a Key Intermediate Point in the Anti-Cancer Progression

HOX proteins interact with specific cofactors to select their downstream binding sites in the genome. In vertebrates, those including pre-B cell leukemia transcription factor (PBX) and mouse heterotopic integration, belong to the tricarboxylic acid cycle family of homologous domain protein. In addition, these HOX cofactors can increase the nuclear translocation of HOX protein from the cytoplasm to the nucleus. Nuclear translocation of HOX protein is inhibited by suppressing the formation of HOX/PBX dimer, which impairs the function of HOX transcript factors (165). Therefore, considering the use of HOX protein as cancer therapeutic targets, its interaction with cofactors needs to be determined. Therapeutic values of HOX factors in human cancer are seen in **Table 2** (4, 7, 13, 14, 17, 18, 22, 23, 25–27, 29, 31, 32, 35, 41, 45, 56, 57, 68, 71, 72, 81, 82, 84, 87, 88, 90, 97, 116, 117, 127, 128, 132–135, 137, 141, 147, 148, 153, 166, 167).

**Table 2:** Therapeutic values of HOX factors in human cancer.

Gene	Down regulation effects	Downstream regulated molecules or pathways	Upstream regulatory molecules	Tumor x cell lines x normal tissues	Related targeting molecules	Intervention therapy	Clinical trial number & Reference
HOXA1	Proliferation, migration, invasion, metastasis	Wiping out epigenetic silencing of HOXA1	HOTAIRM, G9a, EZH2	GBM	Histone modification of H3K9, H3K27	/	(116)
HOXA1	Metastasis, invasion	MITF	TGF-β	Melanoma	TGF-β signaling	/	(97)
HOXA1	Chemoresistance(up)	/	MiR-100	SCLC	HOXA1	/	(22)
HOXA3	Occurrence, development	/	HOXA-AS2, miR-15a-5p	PTCC	HOXA-AS2/miR-15a-5p/HOXA3 axis	/	(118)
HOXA3	Chemoresistance	EMT& Cisplatin resistance	HOXA-A3	NSCLC	HOXA-A3	/	(13)
HOXA4	Growth, migration, invasion	β-catenin, cyclin D1, c-Myc, survivin	GSK3β	Lung cancer	GSK3β	LiCI	(14)
HOXA5	Angiogenesis	/	MiR-130-3p	HCC	SP1/MiR-130-3p/HOXA5	SP1 inhibitor	(22)
HOXA5	Growth, migration, invasion	CAF	Exosomal miR-181d-5p	Breast cancer	HOXA5/CDX2	/	(41)
HOXA5	Arresting cell cycle	TP53, P21	Binding to TAAT motif within promoter of TP53	Cervical cancer	Wnt/β-catenin	/	(84)
HOXA7	Migration, invasion	Snail	/	HCC	HOXA7/Snail	/	(25)
HOXA7	Proliferation, invasion	/	CircSLC26A4	Cervical cancer	QKI/circSLC26A4/miR-1287-5p/HOXA7 axis	/	(81)
HOXA9	Leukemogenesis	STAT5, AP1	JAK3/STAT5	Leukemia	Mutant JAK/STAT/HOXA9	PIM1 kinase	(88)
HOXA9	Leukemogenesis	CEBPα, MLL3, MLL4	HOXA9/Meis	Leukemia	Histone H3K4 methylation	/	(128)
HOXA9	Leukemogenesis	Meis, Syk	MiR-146a	Leukemia	HOXA9/Meis/Syk feedback loop	/	(87)
HOXA9	Leukemogenesis	Erg	Trib1	Leukemia	HOXA9/Trib1/Erg	JQ1	(90)
HOXA9	Regulating glucose metabolism reengineering	HIF1α, HK2, GLUT1, PDK1	Onco-miR-365	CSCC	MiR-365-HOXA9-HIF1α axis	/	(127)
HOXA9	Tumor aggression	HOXA9	BRCA1	Breast cancer	/	/	(132)
HOXA10	Tumor aggression	HOXA10, CCSCs	LINC00355/miR-195	HNSCC	LINC00355	/	(35)
HOXA11	Proliferation, cell-cell adhesion pathway	LncRNA HOXA11-AS, LSD1	EZH2	GC	EZH2/HOXA11-AS/LSD, HOXA11-AS/miR1297/EZH2	/	(56)
HOXA11	Metastasis, invasion	β-catenin, P21, KLF2	WDR5/EZH2/STAU1	GC	HOXA11-AS	/	(57)
HOXA13	Metastasis	ACLY, IGF1R	IGF1	CRC	IGF1R/ACLY	Linsitinib/ETC-1002	(133, NCT01154335, NCT01016860)
HOXB3	Tumor aggression	RASSFIA, DNMT3B	POL2	Lung adenocarcinoma	RASSFIA	RASSFIA epigenetic silencing	(153)
HOXB3	Tumor aggression	CDCA3	/	Primary prostate cancer/PC-3/LNCaP	HOXB3	/	(72)
HOXB4	Arresting cell cycle	β-catenin, TCF, c-Myc	/	Cervical cancer	Wnt/β-catenin	/	(82)
HOXB5	Metastasis	ITGB3, CXCR4	CXCL12	CRC	CXCR4	AMD3100	(134, NCT02179970)
HOXB5	Metastasis	CXCL1, FGFR4	FGF19	HCC	FGFR4	BLU-554	(26, NCT02508467, CT04194801)
HOXB7	Metastasis	TGFβ2, SMADS	/	Breast cancer	MET	/	(135)
HOXB7	Metastasis	c-Myc, Slug	/	HCC	MET	/	(27)
HOXB8	Invasiveness	BACH1	/	CRC	/	/	(137)
HOXB9	Angiogenesis	IL-6, VEGF	/	Colorectal cancer	VEGF	Bevacizumab	(166)
HOXB9	Migration, tumor growth	JMJD6	Ack27	Lung adenocarcinoma	Ack27-HOXB9	/	(23)
HOXB9	Migration, tumor growth	EZH2	Ack27-HOXB9	Clone cancer	Ratio of Ack27-HOXB9/HOXB9	/	(167)
HOXB13	Tumor aggression	/	/	Prostate cancer	HOXB13, G84E variant	/	(71)
HOXB13	Metastasis	RFX6	Rs339331 at 6q22	Prostate cancer	RFX6	/	(68)
HOXB13	Tamoxifen resistance of breast cancer	IL-6	HBXIP	Breast cancer	HBXIP	Aspirin	(45)
HOXB13	Tumor aggression	β-catenin, TCF4, c-Myc	DNMT3B	RCC	DNMT3B-HOXB13-c-Myc axis	/	(141)
HOXC8	Cisplatin chemoresistance, anti-apoptosis	TGFβ1	/	NSCLC	HOXC8	/	(17)
HOXC9	Proliferation, migration	DAPK1-beclin1	/	Glioblastoma	HOXC9/autophagy	/	(4)
HOXC9	Chemoresistance	SRSF2, PLAU, HIC2	MiR-193a-3p	Bladder cancer	MiR-193a-3p/HOXC9/DNA damage	/	(32)
HOXC10	Aberrant expression	HOXC10	PRC2	NSCLC	Kras-mutant/HOXC10	BET/MEK inhibitor	(18)
HOXC10	Angiogenesis	VEGFA	/	Gliomas	HOXC10/VEGFA	Bevacizumab	(7)
HOXC10	Invasiveness	VASP, PDPK1	IL1β	HCC	IL1R1	Anakinra	(29)
HOXD3	Tumor aggression	Integrinβ3	HOXD-AS1	CRC	HOXD-AS1-HOXD3-integrinβ3	/	(147)
HOXD10	Invasion	Snail, Slug, MMP2, MMP9, MMP14, E-cadherin	MiR-23a	Gliblastoma	MiR-23a/HOXD10	/	(148)

Morgan et al. have found HXR9 peptides specifically target the interaction between HOX and PBX (78, 168). HXR9 inhibits HOX function by preventing its binding to PBX, which leads to apoptosis in multiple mouse breast cancer derived cell lines (168). In addition, the interaction between HOX and HXR9 has been shown to cause apoptosis in numerous cancers, including melanoma, mesothelioma, myeloma, renal cancer, prostate cancer, lung cancer, ovarian cancer, pancreatic cancer, squamous cell cancer of the head and neck, and oral cancer (58, 78, 95, 98, 168). Kaspar and Reichert have already used HOX/HXR9-based treatments in cancer treatments, and Morgan and others are exploring a variant of HXR9 for intratumoral injection in clinical trials (168). The HOX gene targeting therapy is also being explored through RNA interference approaches (158). For example, MicroRNAs transcribed in HOX clusters, namely miR10A32 and miR196B33, regulate HOX expression through RNA interference (158). These miRNAs cleave or inhibit the translation of HOX mRNA. The use of these miRNAs in cancer cells *in vitro* seems to modulate HOX gene expression and its effect on cancer progression.

In fact, HOTAIR has the potential to be an effective therapeutic target for many types of cancer, where abnormal HOXD expression has been found to be associated with cancer progressions, such as cancers in breast, stomach, colon, cervix, lung, and liver (11, 12, 24, 36, 52–54, 158, 159, 169, 170). For example, HOTAIR is highly expressed in cervical cancer compared to normal cells (158, 159, 169). However, its knockout in cervical carcinoma cells induces apoptosis and inhibits tumor proliferation, migration, and invasion.

### 3.2 HOX Genes as Therapeutic Targets

Previous studies have shown that HOX protein exerts carcinogenic activity not only through its reversed transcription ability but through its protein interaction network.

When designing domain-specific HOX inhibitors, gene targeting must be taken into account. The problem in bringing monoclonal antibodies into clinical therapies is their nuclear localization. To address the above issue, the focus of next-generation immunotherapies is to develop smaller monoclonal antibody fragments or totally new entities to improve tissue permeability and subcellular localization. These crucial immunotherapeutic strategies are also focused on addressing the treatment of hematologic tumors and solid tumors.

Since HOX protein is closely linked to brain malignancies where the blood brain barrier is a major challenge for drug infiltration, this provides instructive thinking of treatment. Another strategy for targeting oncogenes in cancer cells is to use small interfering RNAs (siRNAs) (171). Many studies have shown inhibition of HOX expression by siRNAs can distinctly retard tumor growth and aggressiveness. Similarly, the use of siRNA strategies may be an effective therapeutic approach for targeting HOX genes *in vivo*.

Although the strategy is not as progressive as small molecular chemical compound inhibitors or monoclonal antibodies, efforts have been made over the past decade to implement it in cancer treatment. At present, poor uptake of cells, side effects of packaging-related methods are major obstacles to HOX targeting clinical application. As a result, current efforts are made to address a range of novel strategies for delivering siRNAs *in vivo*.

The small-molecule chemical inhibitors are of anticancer potential. We highlight the recent therapies targeting HOX genes-downstream proteins especially in HCC and colorectal cancer. Overexpression of HOXB5 transactivates downstream protein expression of FGFR4 and CXCL1, hence promoting HCC metastasis. The small chemical compounds application of FGFR4 inhibitor BLU-554 and CXCR2 inhibitor SB265610 sharply inhibits HCC metastasis mediated by HOXB5 (26). Integrins are also involved in HOX-induced cancer metastasis. In CAOV-3 cells, HOXA4 overexpression inhibited migration and increased the protein level of β1 integrin, suggesting that the β1 integrin may be involved in HOXA4’s inhibitory effect on cell motility (79). In HOXC10-VASP/IL-1R1 mediated HCC metastasis, daily administration of IL-1R1 antagonist anakinra dramatically prolongs survival time (29). In colorectal cancer, the molecule network IGF1-HOXA13-ACLY/IGF1R and CXCL12-HOXB5-CXCR4/ITGB3, targeted blocking the downstream protein with small molecular compounds serves as a promising anticancer therapy (133, 134).

Furthermore, natural and synthetic drugs regulating HOX genes network are associated with anticancer and antibacterial activities. For instance, researchers synthesized the new molecule 5H-pyrido[3,2-a] phenoxazine-5-one in the laboratory, evaluated its ability to regulate the activity of lncRNA HOTAIR and HOXC locus genes from HOXC9 to HOXC13 in MCF-7 human breast cancer cell lines, and confirmed that 5H-pyrido[3,2-a] phenoxazine-5-one was able to inhibit the relevant HOX gene expression and counteract the pathogenesis of breast cancer (172).

The discovery of targeting downstream molecules with chemical compounds has enhanced the potential to specifically target HOX genes intermediated network and eliminate the cancer development, which may improve clinical outcomes in cancers.

## 4 Conclusion

Although it is difficult to intercept various transcription factors, alternative strategies based on the exploitation of the associated molecular networks are emerging. The HOX genes play an important role in cancer progression, showing great plasticity and interfering with many molecular mechanisms. Abnormal HOX expression by altering its homologous box methylation profile, is often associated with cancer. In addition, different HOX gene regulatory features describe different cancer types and are increasingly being used as cancer biomarkers. Their role in cancer progression is based on the ability to control gene expression either directly as transcriptional regulators or indirectly through epigenetic control. In fact, HOX proteins have been shown to be involved in different epigenetic mechanisms involved in DNA and histone methylation, which may be related to the epigenetic regulation of multiple cancer related genes. Therefore, it is very important to seek pharmacological agents that are synthetically lethal in conjunction with HOX overexpression. Therapies targeting HOX molecules should intake the functional redundancy among the different HOX family members, so that appropriate therapeutic combination can be created. There is an urgent need for a more comprehensive understanding of their biology, and identifying their gene targets and molecular networks involved in tumor progression.

## Author Contributions

Conceptualization, YF, and LX. Writing—original draft preparation, YF. Writing—review and editing, YF, TZ, YW, MX, XJ, WH, and LX. Supervision, TZ, MX, XJ, XL, and LX. Project administration, WH and LX. Funding acquisition, WH and LX. All authors contributed to the article and approved the submitted version.

## Funding

The research was supported by grants from the National Natural Science Foundation of China No. 81871911 (WH), No. 81972237 (LX), and No.81772623 (LX), and the National Key Research and Development Program of China 2018YFC1312103 (LX).

## Conflict of Interest

The authors declare that the research was conducted in the absence of any commercial or financial relationships that could be construed as a potential conflict of interest.

## Publisher’s Note

All claims expressed in this article are solely those of the authors and do not necessarily represent those of their affiliated organizations, or those of the publisher, the editors and the reviewers. Any product that may be evaluated in this article, or claim that may be made by its manufacturer, is not guaranteed or endorsed by the publisher.

## Abbreviation

NSCLC, non-small-cell lung carcinoma; CRC, colorectal cancer; PTC, papillary thyroid cancer; LSCC, laryngeal squamous cell cancer; GC, gastric cancer; HCC, hepatocellular carcinoma; FASN, fatty acid synthase; LUAD, lung adenocarcinoma; HNSCC, head and neck squamous cell carcinoma; TNBC, triple negative breast cancer; CMM, cutaneous malignant melanoma; ESCC, esophageal squamous cell carcinoma; NPC, nasopharyngeal carcinoma; CHOL, cholangiocarcinoma.
